# Organic Farming Shapes Population Dynamics and Genetic Diversity of *Euborellia annulipes* in Banana Groves

**DOI:** 10.3390/insects16060606

**Published:** 2025-06-08

**Authors:** Pilar Jurado-Angulo, Mario García-París, Natalia Rosas-Ramos

**Affiliations:** 1 CIBIO—Centro de Investigação em Biodiversidade e Recursos Genéticos, InBIO Laboratório Associado, BIOPOLIS Program in Genomics, Biodiversity and Land Planning, Campus de Vairão, Universidade do Porto, 4485-661 Vairão, Portugal; 2 Departamento de Biologia, Faculdade de Ciências da Universidade do Porto, 4169-007 Porto, Portugal; 3 Departamento de Biodiversidad y Biología Evolutiva, Museo Nacional de Ciencias Naturales (MNCN-CSIC), c/José Gutiérrez Abascal, 2, 28006 Madrid, Spain; 4 Instituto de Engenharias e Ciências do Mar (ISECMAR), Universidade Técnica do Atlântico (UTA), Mindelo 163, São Vicente, Cabo Verde; 5 Departamento de Biología Animal (Área de Zoología), Universidad de Salamanca, 37007 Salamanca, Spain; nataliarosasr@usal.es

**Keywords:** earwigs, agroecological management, mitochondrial variation, traditional agriculture, Canary Islands

## Abstract

Organic farming can help mitigate the negative impacts of agriculture on biodiversity, but its effects remain controversial and poorly understood for many taxa, especially from a genetic perspective, where major knowledge gaps persist. This study investigates how the organic and conventional management of banana groves influences population dynamics (i.e., total abundance and abundance by sex and developmental stage) and mitochondrial genetic diversity (cytochrome b gene) of the earwig *Euborellia annulipes*, a natural biological control agent. The results revealed higher overall abundance, particularly of females, in organic groves. This could be due to females’ more sedentary behaviour linked to parental care, increasing their vulnerability to local disturbances such as pesticide application. In contrast, males, being more mobile, did not respond to the farming system. Genetic analyses revealed similar haplotypic diversity across systems but higher nucleotide diversity in conventional orchards. This may suggest either pesticide-induced mutations associated with detoxification and resistance or increased gene flow driven by greater mobility in response to disturbance. Although the high genetic diversity observed raises questions about the introduced status of *E. annulipes*, its origin remains unresolved. These findings highlight the relevance of integrating ecological and genetic data when assessing the impacts of agricultural practices on beneficial arthropods.

## Simple Summary

Organic farming can contribute to counteract the negative effects of agriculture on biodiversity, but its impact—especially on genetic diversity—is not well understood. This study examines how organic and conventional farming influence the population dynamics and genetic diversity of the earwig *Euborellia annulipes*, a beneficial insect, in banana groves. We found more earwigs in organic orchards, and particularly more females, possibly because they are more sensitive to pesticides due to their sedentary behaviour associated with parental care. Males, being more mobile, showed no difference between management regimes. Genetic analyses revealed that, although both systems exhibited similar levels, conventional groves had a greater nucleotide diversity. This may suggest that pesticide exposure is inducing mutations related to detoxification and resistance, or that increased gene flow—driven by greater mobility in response to disturbances—is enhancing genetic mixing. Our findings illustrate that combining ecological and genetic information is crucial for better understanding how agricultural practices affect invertebrates.

## 1. Introduction

Agricultural biodiversity is currently facing multiple threats, including habitat loss, the homogenisation of agricultural systems, or the intensive application of pesticides and fertilisers [1]. The decline in agrobiodiversity may have significant implications for food security, as it diminishes critical ecological services like natural pest control, consequently increasing the vulnerability of crops to pest outbreaks, diseases, and climate-related shocks [2,3,4]. Nevertheless, agriculture itself is not solely responsible for the significant loss of biodiversity but rather expansion and intensification processes [5,6,7]. In fact, certain forms of agriculture and management strategies, such as traditional farming, agroforestry, low-intensity management, crop rotation, or the preservation and promotion of habitat heterogeneity, make agroecosystems sustainable environments capable of supporting high levels of biodiversity [8,9,10,11,12].

Another strategy that enables the establishment of ecologically balanced (i.e., resilient, diverse) and productive ecosystems is organic farming. This low-intensity system adheres to rigorous guidelines and regulations that, among the key provisions, prohibit the application of synthetic pesticides and fertilisers, the use of genetically modified organisms (GMOs), and boost the promotion of functional biodiversity for enhancing ecosystem services (Regulation UE 2018/848). From a biodiversity perspective, there is some controversy about the effects of organic farming on biodiversity [11]. Overall, it is considered that wildlife-friendly practices developed under organic management can help to counteract the negative impacts of agriculture on associated biodiversity [7,13,14,15,16,17,18,19]. However, it is also emphasised that the benefits of organic farming can be contingent upon factors such as geographical region, crop type, management practices, or the composition of the species or taxa involved, thus resulting in heterogeneous impacts on biodiversity [1,14,15,20,21,22,23].

Agrobiodiversity comprises different components [24]. In fact, agricultural zones often constitute the habitat of non-native species that have been introduced and spread across cropped areas worldwide by changing plant or soil material [25,26,27], a process that is currently being magnified by the increase in global trade [28,29]. Although most non-native insects stand out for their global negative impacts [30,31], other have been deliberately introduced due to their functional role in order to enhance ecosystem services such as biological control [32,33].

Ecosystem homogenisation tends to simplify the genetic diversity of species that inhabit them, as the reduction in ecological niche variety simultaneously reduces the range of selective pressures promoting genetic diversity within populations [34,35]. Agricultural intensification, which generally involves a homogenisation of agricultural systems at both local and landscape levels, may lead to a reduction in the genetic diversity of genotypes [36,37]. It is noteworthy that, although genetic diversity determines the evolutionary potential of a species, the extent to which agricultural practices influence such diversity remains poorly understood. This genetic footprint, shaped by agricultural practices, may also interact with that left by species introductions. Populations introduced to new environments, typically derived from a small number of individuals (founder effect), tend to experience a reduction in genetic diversity compared to their native counterparts [38,39].

**Figure 1:**
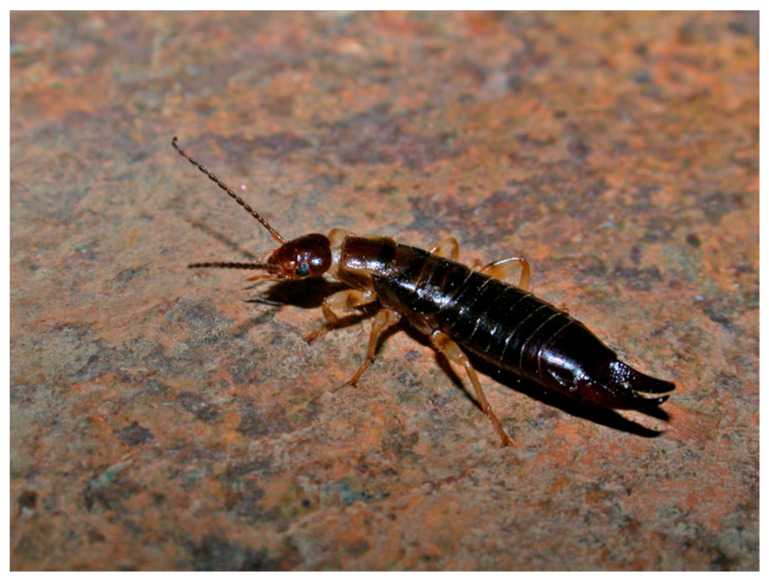
Female *Euborellia annulipes* (Lucas, 1847) from Puntallana, La Palma, Canary Islands. Photograph by M. G.-P.

Among the multiple examples of alien species, earwigs, considered to be significant biocontrol agents [1,40,41,42], have been frequently introduced worldwide through the international trade of plants and goods [25,43,44,45,46,47,48,49,50]. The introduction of species of the genus *Euborellia* Burr, 1910 has been reported in many European, American, and Australasian countries, sometimes as a biological control agent [25,44,46,50,51,52,53,54]. Occasionally, some species were described from greenhouses where they were already introduced, rendering the origin of these species doubtful, as is the case of *E. arcanum* Matzke and Kočárek, 2015 or *E. annulipe*s (Lucas, 1847). *Euborellia annulipes* (Figure 1), possibly native to the Mediterranean or East Africa [50,54], is considered a “probable introduced species” by the Canary Islands Government [55], where it is extensively present in cropped areas.

In the Canary Islands, agriculture plays an important role in shaping the islands’ landscape, especially the banana production [56,57,58]. Banana crops hold substantial global significance, ranking as the world’s 12th largest crop in terms of production [59]. In the Canary Islands, banana production constitutes approximately 30% of the total agricultural output [57,60], making it the primary agricultural crop in the region and an essential part of the economy and culture of the archipelago. Although numerous farmers have transitioned to organic farming practices, in global terms, there is limited available information on the impact of organic banana cultivation on biodiversity, even considering that biodiversity responses to organic farming can vary widely among crop types [14].

In this study, we examine populations of *E. annulipes* in banana crops on the island of La Palma from an ecological and genetic perspective in order to (i) evaluate the extent to which farming system (organic vs. conventional) determines the overall abundance of the species, (ii) analyse whether potential sensitivity to farming system differs between sexes and/or instars, (iii) assess whether the genetic diversity of the cytochrome b mitochondrial gene (cytb) of *E. annulipes* is influenced by farming system, and (iv) explore the ecological or historical factors that may have shaped these patterns of genetic diversity in *E. annulipes*.

## 2. Materials and Methods

### 2.1. Study Area

This study was carried out in 2021 in Breña Baja and Puntallana on the island of La Palma (28°38′36″ N 17°46′7″ W; 28°45′38″ N 17°44′47″ W) (Canary Islands, Spain). La Palma is located in the northwestern area of the Canaries, with an area of 728 km^2^ and a maximum altitude of 2426 m a.s.l. [61]. The climate is predominantly subtropical–Mediterranean, with humid winters and dry summers. However, climatic conditions differ considerably within the island. Annual precipitation ranges from about 170 mm to almost 1400 mm, although fog drip can locally lead to an increase in precipitation, particularly during the summer. The north and east slopes of the island are especially exposed to the trade winds below 1500 m, which make them extremely humid. The annual temperature ranges from about 9 °C on the island summit to around 22 °C at the leeward southwestern coast [62].

The zonal vegetation reflects the climatic conditions of the island, including halophytic communities in arid coastal areas, succulent scrub and thermophilic woodlands in semi-arid lower elevations, endemic Canary Pine Forest in mid-elevations, or high-elevation summit scrub [62].

Lowlands are dominated by traditional banana crops (*Musa acuminata* Colla) that, due to the rough topography of the island, consist of small fields arranged on terraces. However, other crops such as avocado (*Persea americana* Mill) and vineyard (*Vitis vinifera* L.) are also well represented in the area [63]. Traditional orchards are interspersed with remnants of natural vegetation, shaping a mosaic agricultural landscape. These remnants of natural vegetation are formed mainly by sub-desert shrubby vegetation, typically present in the low-elevation parts of the island, and composed of singular species of *Euphorbia* L., *Senecio* L., and *Aeonium* Webb & Berthel, among others [64].

### 2.2. Sampling Design

Across a study area of 170,000 and 500,000 m^2^, we sampled banana groves under different management regimes (three conventional and three organic groves) in an elevation range of 75 to 200 m. Organic and conventional groves were not clustered in separate zones but were interspersed throughout the landscape. The mean field area was 2017 ± 303 m^2^, and the mean nearest-neighbour distance was 647.82 ± 191.7 m. Organic banana groves were certified for organic production (EU Parliament Regulation 2018/848) [65], which implies that no synthetic fertilisers and pesticides are applied. Among the management strategies implemented in organic farming is the application of fertilisers made from organic plant and animal matter and manure, such as enriched liquid manure and compost [66,67]. Banana exhibits vegetative propagation and is mostly grown as a perennial crop. Propagation traditionally consists of selecting plant sprouts from the mother plants growing in the field. Banana plants are periodically renewed by cutting old plants, which are replaced by newly selected plants [68,69]. Once cut, old stems are typically left in the soil within the plot.

Earwigs, *Euborellia annulipes*, were sampled inside the cut banana stems of the six groves in June over five consecutive days. In each banana grove, we randomly selected a total of eight cut stems of similar size (approximately 20 cm in diameter) and at an advanced stage of decomposition in order to ensure comparable microhabitat conditions. Furthermore, all stems were shaded similarly, avoiding differences in temperature and humidity. All sampling was conducted during the same daytime interval (12:00–17:00), a period when earwigs shelter in the stems. The stems were thoroughly inspected for *E. annulipes* by shredding the rotten tissue; all specimens detected were taken directly by hand and transferred to individual 2 mL cryotubes with absolute ethanol for preservation. Sex and instar were determined for all the collected individuals.

### 2.3. Statistical Analysis

We applied separate generalised linear models (GLMs) to analyse the effects of farming system (organic vs. conventional farming) on (i) total earwig abundance and on the number of (ii) adults (female plus male), (iii) nymphs, (iv) females, and (v) males. Models were fitted with a Poisson distribution and log link function and were inspected for overdispersion. When overdispersion was detected, the models were adjusted with a negative binomial distribution. All models were validated by inspecting the residuals both graphically and with the DHARMa package. Analyses were performed using R 4.3.1 software [70].

### 2.4. DNA Extraction, Amplification, and Sequencing

We obtained total DNA from 12 specimens of *E. annulipes* (2 specimens from each banana grove) (Table 1). DNA was extracted from one leg, using the DNeasy Blood and Tissue Isolation Kit (Qiagen, Hilden, Germany), according to the manufacturer’s instructions, and then stored at 4 °C until further processing. The polymerase chain reaction (PCR) was used to amplify fragments (385 bp, 373 bp after end trimming) of the cytochrome b gene (cytb) using the set of primers CB-J-10933 (TATGTACTACCATGAGGACAAATATC) [71] and CB4 (AAAAGAAARTATCATTCAGGTTGAAT) [72]. PCR amplifications consisted of 18.8 μL of distilled water, 2.5 μL of 10× PCR buffer, 1 μL of dNTP mix (10 mM), 0.5 μL of MgCl_2_ (50 mM), 0.5 μL of each primer (10 μM), 0.2 μL of DNA polymerase (5 U/μL), and 1 μL of DNA template, consisting of a final reaction volume of 25 μL. The thermocycling conditions consisted of an initial denaturation at 95 °C for 5 min, followed by 40 cycles of denaturation at 94 °C for 1 min, annealing at 40 °C for 1 min and extension at 72 °C for 1 min, and a final elongation step at 72 °C for 5 min. After the amplification, 3 μL of the reaction was analysed by electrophoresis for 40 min at 90 V on a 1% agarose gel. Samples with single bands were sent to the company Macrogen Inc. (Macrogen Europe, Madrid, Spain) for sequencing both strands.

Sequences were checked, edited, and aligned using Geneious Prime 21.1.1 software.

### 2.5. Genetic Analyses

To identify potential patterns of cytb variation driven by the farming system (conventional vs. organic), haplotype networks were generated with Population Analysis with Reticulated Trees (PopART), using a TCS algorithm to shape the relationships between haplotypes. Haplotype networks were edited with Adobe Photoshop CS5 version 12.0 (Adobe Systems Incorporated). Uncorrected (*p*) pairwise genetic distances were estimated using PAUP * v.4.0a [73].

Genetic diversity in populations of *E. annulipes* from both organic and conventional groves was analysed by calculating the haplotype diversity (Hd) and nucleotide diversity (π) of cytb sequences. Both metrics were computed using R 4.3.1 software [72]. Sequences were categorised into two groups based on the farming system (organic and conventional), and diversity analyses were conducted using the ‘pegas’ [74] and ‘poppr’ packages [75]. Haplotype diversity reflects the probability that two randomly chosen sequences are different, i.e., it represents the variety of haplotypes present. Nucleotide diversity estimates the average number of nucleotide differences between pairs of sequences, i.e., it considers how different the sequences are from each other.

## 3. Results

### 3.1. Effects of Farming System on the Abundance of Euborellia annulipes

A total of 218 specimens of *E. annulipes* were collected from banana stems, of which 121 were adults, 97 were nymphs, 84 were females, and 37 were males.

**Figure 2:**
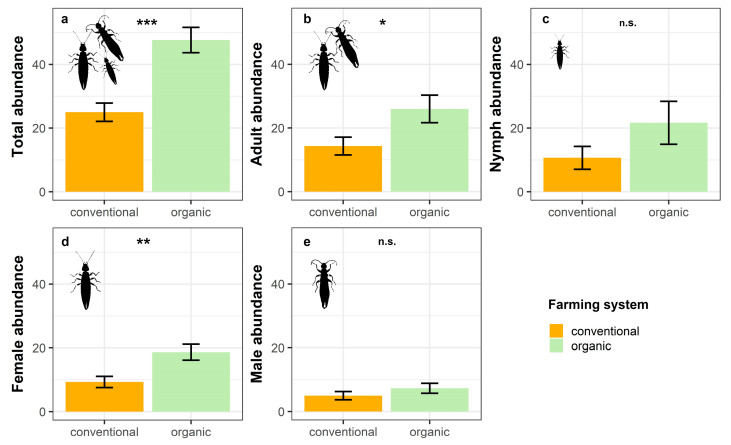
Estimated mean ± SE of (**a**) total abundance, (**b**) adult abundance, (**c**) nymph abundance, (**d**) female abundance, and (**e**) male abundance of *E. annulipes* in conventional and organic banana groves (n.s. not significant, * *p* < 0.05, ** *p* < 0.01, *** *p* < 0.001).

The results from the GLMs showed that total earwig abundance was significantly affected by the farming system, with organic banana groves harbouring larger numbers of *E. annullipes* than conventional ones (Table 2; Figure 2a). However, the benefits of organic farming differed between sexes and also varied depending on the development stage.

Thus, adults were benefited by organic farming, whereas nymphs did not show any significant response to this factor (Table 2; Figure 2b,c). Regarding sexes, females reached significantly higher abundances in organic orchards compared to conventional orchards (Table 2; Figure 2d). Conversely, we did not find any effect of farming system on males, with their densities being similar under both management regimes (Table 2; Figure 2e).

### 3.2. Genetic Diversity

**Figure 3:**
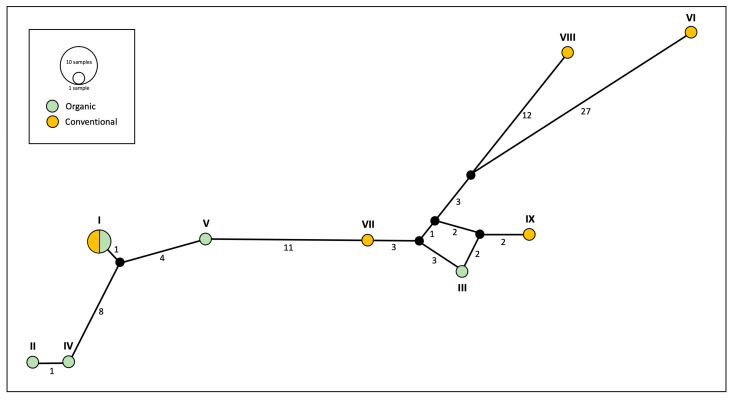
Mitochondrial (cytb) network analyses of specimens of *Euborellia annulipes* from organic and conventional banana groves. Numbers below the lines connecting haplotypes represent the number of mutations from one point to another. Roman numerals (I–IX) refer to the haplotype assignment for each specimen (see Table 1).

The phylogeographic network based on the mitochondrial gene cytb (Figure 3) revealed nine distinct haplotypes among the 12 specimens of *E. annulipes* analysed. One haplotype was shared between the two farming systems, occurring in two individuals from organic groves and two others from conventional orchards. The remaining eight haplotypes were evenly distributed, with four exclusively present in organic farming and the other four unique to conventional management.

Sequence divergence within mtDNA from organic banana groves was relatively low (uncorrected *p* distance = 0.0000–0.0643). In comparison, genetic distances within conventional (*p* distance = 0.0000–0.1206) and across samples from conventional and organic orchards (*p* distance = 0.0000–0.1260) were considerably large. Genetic diversity analyses of cytb sequences highlighted differences between the two farming systems in terms of nucleotide diversity (π). Sequences from conventional banana groves exhibited significantly higher nucleotide diversity (π = 0.070) compared to those from organic ones (π = 0.033). This is consistent with the results from the genetic distance matrix, where we observed that genetic distance between sequences within the conventional orchards was significantly higher than the distance between sequences within organic groves. In contrast, haplotype diversity was identical across both management regimes (Hd = 0.933), consistent with the results from the haplotype network (Figure 3), where an equal number of unique haplotypes was identified in each system.

## 4. Discussion

The discussion surrounding the impact of agricultural systems on organisms’ population structure continues to advance. Organic farming, in particular, appears to create conditions that benefit certain species, though their responses can vary significantly. Consistent with previous studies on diverse earwigs [76,77,78], the total abundance of *E*. *annulipes* was higher in organic groves compared to conventional ones. Studies on *Forficula auricularia* Linnaeus, 1758 have revealed that more complex and less intensive agricultural systems, such as organic farming, tend to support higher populations, likely as a result of increased food resources and shelter availability [79,80]. In addition, pesticide application inevitably involves the exposure of non-target organisms, with consequent effects on species, communities, or ecosystems [81]. Among that, earwigs are sensitive to pesticide application and can also be influenced by different agricultural practices and by crop characteristics [22,81,82,83]. Therefore, the ban of pesticide use in organic farming can promote higher survival rates of beneficial fauna, including earwigs [76,80], which could explain the observed greater abundance of *E. annulipes* in these systems. However, earwig responses to organic farming are not always consistent [22,78,82], as we detected when diving deeper into how the different sexes and life stages are influenced by the management regime.

Aligning with previous studies that demonstrate sex-specific reactions to agricultural management [80,82,83], we found that males and females exhibited contrasting patterns, with only females being favoured by organic farming. This disparity may be linked to the differing behaviours of both sexes and their distinct biological roles. Females of *E. annulipes* provide intensive parental care to the eggs, but males provide no care and frequently prey upon them [84]. During offspring development, females take care of the eggs, licking and cleaning them to prevent fungal infections [85,86], and also care for nymphs during the first days of development [87]. This behaviour suggests that females exhibit a high fidelity to a specific microhabitat, being largely conditioned by the local characteristics of that habitat since they remain close to the nest. In the specific case of conventional banana groves, this situation could lead to greater exposure to chemicals, and thus to a reduced abundance in such systems. This hypothesis is consistent with previous studies demonstrating that females can be more sensitive to pesticides than males [88]. In contrast to the females, males of *E. annulipes* are likely to be generally more mobile, spending more time searching for mates or food [84,85,86]. Their increased mobility may render them less susceptible to local habitat characteristics, enabling them to seek refuge in areas with reduced pesticide exposure. Our results suggest that male biology or behaviour may confer resilience to certain environmental pressures, which could explain their consistent abundance across both organic and conventional orchards.

These findings could have implications for key ecosystem services, such as crop pest control. Most earwigs are known as generalist predators that capture a wide variety of prey [53,89] and may play an important role in controlling orchard pests in the absence of chemical pesticides [77,90,91], as occurs in organic farming. In the specific case of our focus species, due to its high voracity preying upon several insect orders, such as Diptera [92,93], Hemiptera [94], Lepidoptera [95,96], and Coleoptera [85], *E*. *annulipes* has been considered a promising natural enemy [97,98,99,100]. In addition, Coelho et al. [97] found that females can consume larger numbers of prey than males. In view of the above considerations, the situation depicted by our results could imply that pest control may be to some extent reinforced in organic banana groves, in which not only higher densities of *E*. *annulipes* but also larger numbers of females were recorded compared to conventional ones.

In relation to life stages, although adults were overall favoured by organic farming, we did not detect any significant differences in nymph preferences. This observation is striking considering that in experiments testing pesticide impacts where different instars have been used, adult earwigs were found to be less sensitive than nymphs [88,98,101,102]. In addition, Meunier et al. [103] reported that pesticides exert sublethal effects on *F. auricularia* females, particularly affecting maternal care behaviour; females exposed to pesticides tended to abandon their nests for prolonged periods, with significant reductions in their grooming and egg-clumping activities. As previously discussed, females may be somewhat more sensitive to pesticides than males and, at first glance, one might expect these potential effects on maternal care to have a greater impact on egg or nymph mortality. However, this expectation does not align with our findings, which showed no significant differences in nymph abundance between organic and conventional groves. Although some studies suggest that nymphs can be more susceptible to pesticide exposure due to the higher permeability of their cuticles, as noted by [82], it is important to mention that previous research has mainly focused on *F. auricularia*, and specific data on the cuticle permeability of *E. annulipes* are currently lacking.

One potential explanation for the absence of differences in nymph abundance between farming systems could be that, overall, organic farming may promote both biodiversity and predator richness [23,104,105,106], resulting in increased predation pressures. This situation may buffer the potential higher survival rates of nymphs in the absence of pesticides due to a higher predation risk. This hypothesis is consistent with the findings of Sinclair et al. [107], who showed that predation pressure tends to be higher in more biodiverse environments.

In addition to population abundance, genetic diversity provides relevant insights for understanding how populations adapt and maintain resilience to environmental changes [108]. Organic and conventional farming exert different pressures, potentially shaping genetic variation within populations. Habitat heterogeneity and population size are key factors influencing genetic diversity [109], which in turn affects how species like *Euborellia annulipes* respond to agricultural management. In organic farming, one might expect increased species diversity [13,19,110] to be reflected in greater genetic diversity within populations. However, our findings revealed a contrasting pattern: although haplotypic diversity is similar in both systems, conventional groves exhibited higher nucleotide diversity than those under organic management. Furthermore, the genetic distance between conventional orchards was comparable to that observed across different farming systems (0–0.1206 vs. 0–0.1260, respectively). One possible explanation for the larger genetic diversity observed in conventional farming could be related to the selective pressure exerted by pesticides. Previous research has shown that invertebrates exposed to pesticides can develop genetic resistance mechanisms [111]. In the case of *F. auricularia*, for example, pesticides have been linked to mutations in genes associated with detoxification and resistance to these compounds [111]. This adaptive process, driven by a continuous exposure to pesticides, could be increasing genetic variability in *E. annulipes* populations in conventional systems. In addition, conventional banana plantations also tend to be exposed to greater landscape fragmentation and soil changes, which, combined with the presence of chemicals, would result in the invertebrate populations that inhabit them being subjected to a more dynamic and stressful environment. This context could favour a larger divergence between haplotypes. In contrast, being free of harsh pesticides, organic groves may represent more stable environments, in which reduced rates of persistence of new genetic variants may occur due to lower selective pressures. While overall biodiversity (i.e., species diversity) tends to be higher in organic farming [7,16,20,104], this does not have to translate into larger genetic diversity within populations, as we observed in this study.

Another factor that may explain the high genetic diversity in conventional farming is increased gene flow, reflecting a greater contribution of genetic material. This could result from the movement or introduction of specimens, potentially associated with higher mobility in intensively managed systems. A history of agricultural practices that facilitated the introduction of populations from diverse sources may have further contributed to the observed genetic variation.

Genetic variability often plays a crucial role in determining whether a species is native to a region or has been introduced. In native species, genetic diversity tends to reflect long-term evolutionary processes such as local adaptation, isolation by distance, and historical populations’ connectivity. In contrast, non-native species may show signs of recent bottlenecks, founder effects, or genetic homogeneity, particularly when introductions are recent or derived from a limited number of individuals [112,113]. When a limited number of species are studied or in a limited geographic area, high genetic diversity is often associated with native species or species that have been established for a long period of time in the territory. This is because they have had enough time to evolve and accumulate haplotypic diversity. According to our results, we observed high genetic diversity comparable to that reported for other long-established species of earwigs using the same molecular marker (cytb) [114]. Specifically, populations of *Pseudochelidura cantabrica* Cuesta-Segura, Jurado-Angulo & García-París, 2023, from Burgos and León populations of the Iberian Peninsula that are about 100 km apart and separated by mountains of the Cantabrian Mountains), presented an uncorrected mitochondrial (*p*) pairwise genetic distance of 0.112–0.123, comparable to the 0–0.1206 distance presented across populations of *E. annulipes* in this study. These results could suggest that *E. annulipes* has been established for a long time, at least on the island of La Palma. However, *E. annulipes* is currently considered an introduced species by the Canary Islands government [115]. If the species responds to this last hypothesis, the high haplotypic diversity observed may result from a large genetic pool generated through repeated introductions due to the transfer of materials or soil for cultivation. Nevertheless, large populations also tend to exhibit greater genetic variation due to a combination of factors, including higher potential for mutation, a greater probability of retaining new alleles, and a reduced influence of genetic drift. Nevertheless, in this study we have focused on the overall abundance of *E. annulipes*, which serves as a proxy for general population size, rather than examining the effective population size (Ne). It is the effective population size that more directly affects genetic diversity, as it represents the number of individuals that actually genetically contribute to the next generation.

The problem of the origin of *E. annulipes* is far from solved. The native or introduced status in the Canary Islands and other Atlantic Islands is totally unclear. The species was described originally from the greenhouses of the *Jardin des Plantes* of Paris, where according to Lucas [116], the species was introduced in exotic plants brought to the greenhouses, maybe from tropical America. If this was so, the plants were likely stationed in the Canary Islands for some time before reaching the European continent, thus making possible the landing of *Euborellia* in the Islands, or alternatively the boarding of the native earwigs while watering the plants on the ships, if they were native to the Canaries. No molecular studies have been undertaken to study *E. annulipes* populations from the Mediterranean basin, confined to mild-winter sea shores, and compare their genetic structure and diversity with that of populations from the Atlantic Islands and even from the known American populations, which include, for example, high elevation urban areas in inland Mexico (own data). The relatively limited range of the species in Southern Europe and Northwestern Africa might question a Mediterranean origin for the species, while the large diversity shown in our study in cropped areas from a single Atlantic Island (La Palma) does not support, a priori, the introduced status for the species. Even the taxonomy of *E. annulipes* as a whole is questionable, since no direct comparisons of the specimens used in the original description (certainly introduced in Paris) have been established with Mediterranean, Macaronesian, or continental American populations. And the problem is becoming even more complex with the recent discovery of exotic species of *Euborellia* in Europe that can be confused superficially with *E. annulipes* [25,50].

## Figures and Tables

**Table 1:** Specimens of *Euborellia annulipes* (Lucas, 1847) used for DNA analyses with their corresponding voucher numbers and GenBank accession number and haplotype codes.

Specimen Code	Management	Locality	Nº Haplotype	GenBank Cytb
tij21135	Organic	La Palma: El Granel	I	PV737866
tij21136	Organic	La Palma: El Granel	II	PV737867
tij21161	Organic	La Palma: Breña Baja	III	PV737868
tij21162	Organic	La Palma: Breña Baja	IV	PV737869
tij21228	Organic	La Palma: El Granel	V	PV737870
tij21229	Organic	La Palma: El Granel	I	PV737871
tij21281	Conventional	La Palma: La Polvacera	VI	PV737872
tij21282	Conventional	La Palma: La Polvacera	VII	PV737873
tij21324	Conventional	La Palma: Puntallana	I	PV737874
tij21325	Conventional	La Palma: Puntallana	VIII	PV737875
tij21342	Conventional	La Palma: Los Cancajos	IX	PV737876
tij21343	Conventional	La Palma: Los Cancajos	I	PV737877

**Table 2:** Parameter estimates for the generalised linear models (GLMs) assessing the effect of the farming system (organic vs. conventional) on earwig total abundance and on the number of adults, nymphs, females, and males. Reference coefficient is system (conventional) (* *p* < 0.05, ** *p* < 0.01, *** *p* < 0.001).

Response Variable	Factor	Estimate	Std. Error	*z* Value	*p* Value	
Total abundance	Intercept	3.219	0.116	27.876	<0.001	***
(GLM_poisson_)	System (organic)	0.645	0.143	4.527	<0.001	***
Adult abundance	Intercept	2.663	0.195	13.627	<0.001	***
(GLM_nb_)	System (organic)	0.596	0.257	2.319	0.020	*
Nymph abundance	Intercept	2.367	0.336	7.044	<0.001	***
(GLM_nb_)	System (organic)	0.709	0.458	1.546	0.122	
Female abundance	Intercept	2.234	0.189	11.819	<0.001	***
(GLM_poisson_)	System (organic)	0.693	0.232	2.995	0.003	**
Male abundance	Intercept	1.609	0.258	6.233	<0.001	***
(GLM_poisson_)	System (organic)	0.383	0.335	1.144	0.253	

## Data Availability

The genetic data supporting the findings of this study are freely available in GenBank (PV737866- PV737877).
